# Statistical Report on the Sickness, Mortality, and Invaliding among the Troops in the West Indies

**Published:** 1839-07

**Authors:** 


### ART. X.

1. *Statistical Report on the Sickness, Mortality, and Invaliding among the Troops in the West Indies. Prepared from the Records of the Army Medical Department and War-office Returns.* Presented to both Houses of Parliament by Command of Her Majesty.—*London,* 1838. Pp. 103, and Appendix, pp. 40.
2. *Statistical Reports of the Sickness, Mortality, and Invaliding among the Troops in the United Kingdom, the Mediterranean, and British America. Prepared from the Records of the Army Medical Department and War-office Returns.* Presented to both Houses of Parliament by Command of Her Majesty.—*London,* 1839. Pp. 34, 92, and 57.
3. *A Letter to the Right Honorable the Secretary-at-War, on Sickness and Mortality in the West Indies, being a Review of Captain Tulloch's Statistical Report.* By Sir ANDREW HALLIDAY, M.D. F.R.S.E, Deputy Inspector-General of Army Hospitals.—*London,* 1839. Pp. 63.

THE first Statistical Report of Major Tulloch was undertaken for the purpose of fulfilling an enquiry suggested by the secretary-at-war into the extent and causes of the sickness and mortality among the troops in the West Indies, with a view to measures likely to diminish the great loss of life annually experienced in these colonies. It consists mainly and essentially of a digest of the portion relating to the West Indies of the Historical Record of the Medical Transactions of the British Army, which the country owes to the method and industry of the present enlightened director-general of the army medical department, Sir James M'Grigor. The reports in the Medical Board Office have been collated with the War-office returns, and thus a source of inaccuracy, from death so sudden that the patient had not been dieted in hospital, has been obviated, and likewise the average number of men constantly sick has been ascertained. From these conjoint quarters, Major Tulloch, assisted in the first instance by Mr. Marshall, and on the departure of this able statist from London, by assistant-surgeon Balfour, of the medical staff at Chatham, has compiled a statistical report, which, whether we regard the perspicuity, copiousness, and accuracy of the details, or the prudence of the deductions from them, is not surpassed by anything in the records of our art, and which we regard as equally creditable to the compilers, to the science and arrangement of the department whence the principal information was derived, and to the benevolent and patriotic motives of the secretary-at-war, Lord Howick, by whose direction the work was undertaken. It comprises the substance of the reports from the different West-Indian stations during a period of twenty years, viz. from 1817 to 1836 inclusive. The second Report, or that relating to sickness and mortality among the troops in the United Kingdom, is less comprehensive, being necessarily confined to the seven years subsequent to January, 1830; the annual regimental returns furnished to the war-office, whence the sudden deaths, and those which took place on furlough must be gathered, having only then been established. The Reports, however, from the Mediterranean and British America, comprehend the same period of time as that relative to the West Indies, and all are from the same hands, and display the same scientific method and accuracy which are so manifest in the West Indian Report.

The letter of Sir A. Halliday is intended to be at once a critique of Captain Tulloch's Report, and a Supplement to it,—supplementary of that portion of information relative to sickness and mortality in the West Indies which the Report does not profess to give, that, to wit, "which cannot be discovered by the test of facts and figures, and cannot be made the subject of calculation." Though we consider the tone of this pamphlet as often needlessly peevish, yet, knowing it to be the work of a man of talent and experience, we shall embody in our review much of the information it imparts, protesting, however, *in limine*, against the author's professed reliance on opinions relative to the origin of disease not derived from what we must take leave to consider the only sound basis of reasoning on the subject—facts and figures. It is certainly quite supposable that any medical man might have been more lavish of deductions from statistical details coupled with geological and other facts than Major Tulloch has shown himself to be; and it is the fact that Sir A. Halliday has in this pamphlet drawn conclusions from the amount of sickness and mortality in the West Indies, connected with facts which fell under his observation there, which are left untouched in the former gentleman's Report; but on examining these conclusions, we find that figures lie at the foundation of all of them, and that Sir A. Halliday has spoken in a disparaging tone of arithmetic as a ground of reasoning, which his success in employing it has shown to be unmerited. Major Tulloch's Reports being chiefly in a tabular form, and such portions of them as are employed as a commentary on the tables being written as succinctly as possible, a complete analysis of it would be little less than a transcript, and we shall consequently abstain from an attempt to condense further what already exists in the extreme of condensation. We consider, however, that we shall be doing justice to these documents and our readers by endeavouring to gather from them a portion of their more general facts, with certain of the author's deductions, which always bear the stamp of great prudence and circumspection. So far as it appears that inferences, not touched upon by Major Tulloch, are legitimately deducible from this vast repository of facts, of the nature of general truths or principles having a relation to disease wheresoever and in what class soever it prevails; and especially should they refer, either in the way of correction or confirmation, to opinions so generally received as to be considered as recognized medical doctrines, we shall not hesitate to deduce them.

The first Report presents the multitudinous statistical details comprised in it, arranged as they occur, in

1, The Windward and Leeward Command,
2, The Jamaica Command,
3, The Bahamas, and
4, The Honduras Command.

The details of the sickness and mortality in each command are preceded by a concise but, as it appears to us, very clear and accurate account of the geology and meteorology of the district in which they occurred, whilst, besides this general view, the nature of the soil and the general character of the various localities in which the troops are immediately stationed, are described.

In the colonies comprised in the Windward and Leeward Island Command, the mean temperature is about 80½°, and its extreme range is only 13°, whilst in some islands it is only 4° throughout the year. The range of the barometer is not more than from about a quarter to half an inch throughout the year, and the quantity of rain that falls is at least three times as great as in Britain, being estimated at from sixty to seventy inches annually. In this command the admissions into hospital from among the white troops amounted to 1,903 per thousand; so that, on an average, every man must have been under medical treatment once in every six months and a half, while the deaths in the same class, *in hospital*, amounted to 78·5 per thousand. Some corrections of this estimate, required by the occurrence of sudden and accidental deaths, and a discrepancy between the medical and war-office returns of the number of troops in the command, raise the proportion of deaths to 93½ per thousand; or, in other words, the eleventh part of the force has died annually. The mortality among troops in the United Kingdom being fifteen per thousand annually, it follows that the mortality is six times as great in the former situation as in the latter, though the extent of sickness, as shown by the hospital returns, has only been twice as great, an evidence of the much greater severity of the disease in the one situation than in the other.

In Jamaica, from the variety of plain and mountainous surface, almost every diversity of climate may be found. At Kingston, situated on the plain, the maximum of heat is 85°, the mean 80½°, and the minimum 76½°, on an average of all the months throughout the year; whilst at Maroon Town, the highest station occupied by our troops, the maximum is 80½°, the medium 74½°, and the minimum 68½°, likewise on an average of months throughout the year. The quantity of rain which falls annually is fifty inches. In this island the mortality (after all due corrections) is found to be 143 per thousand; or a seventh part of the force has died annually. The four most healthy years were 1823, 1828, 1829, and 1836, when the deaths averaged only 67 per thousand, whilst the most unhealthy were, 1819, 1822, 1825, and 1827, when the deaths averaged 259 per thousand annually. The average being thus during the most healthy period four or five times as great as among troops in Britain, and in unhealthy years nearly sixteen times as great. As an instance of the extreme insalubrity of Jamaica, Major Tulloch mentions that, in the seventeenth century, of 800 troops who arrived in the island, two thirds died in a fortnight.

In Bahamas the maximum temperature (taken, as in the former instance, on an average of months throughout the year,) is 83½°, the mean 78½°, and the minimum 74°. A considerable quantity of rain falls, but the reporter is not acquainted with the exact measurement of it. The admissions into hospital have been 1,430 per thousand annually, and the deaths 200 per thousand, or a fifth of the whole force. This extraordinary mortality, above thirteen times as great as among troops in the United Kingdom, Major Tulloch is inclined to ascribe rather to the unhealthy site of Fort Charlotte Barrack, where the troops are stationed, than to the general insalubrity of the climate, finding that, of the white population of the town of Nassau, only 66 per thousand die annually, being only thrice the rate which prevails in Great Britain.

In Honduras, the average maximum of the thermometer is 81½°, medium 79¼°, and minimum 77°. It rains during five months of the year, and the quantity that falls is very considerable, but it has not been specifically measured. The average annual ratio of admissions has been 1,209 per thousand and 103 deaths, a ratio intermediate between the Windward and Leeward Command and Jamaica. Major Tulloch, however, accords with the current opinion, that this station is more favorable to the health of Europeans than most in the West Indies, finding that the returns of mortality have been swollen by an extremely fatal fever which prevailed in the settlement in 1826.

The main source of this frightful mortality among the white troops throughout these colonies is one form or other of fever, especially the remittent, and that species of disease which is sometimes included in this class, and sometimes mentioned apart, according to the peculiar views of medical men, *yellow fever;* for whilst intermittent and certain other fevers contribute largely to the admissions into hospital, the mortality from them is small. We take the relative mortality from these fevers in the two larger commands to illustrate this point. In the Windward and Leeward Command, whilst intermittents of all types are fatal to one in 159 attacked, remittents are so in the proportion of one in nine, and yellow fever in that of one in one and two thirds. In Jamaica, whilst intermittents are fatal to one in 165, and synochus only to one in 448, remittents destroy one in eight, and yellow fever one in one and a third. When it is considered that the number admitted under the head of remittents is very large in the returns, they constituting nearly one third of the admissions from fever in the Windward and Leeward station, and considerably above four fifths in the Jamaica returns, the reader will reach the conclusion that it must be a main instrument of the fatality of these colonies, without our copying all the statistical details by which it is demonstrated.

The author applies himself to the investigation of the causes producing these fatal diseases; but rather, to our mind, with the effect of showing that medical men have much to reconsider in their existing opinions (a valuable effect, we would remark,) than of establishing any decisive conclusion of his own. Heat presents itself as a probable cause, but he objects to this, that in Antigua and Barbadoes, where the range of the thermometer is rather higher than in Dominica, Tobago, Jamaica, or the Bahamas, the sickness amounts to little more than one third of its prevalence in the latter stations. The prevalence, too, of epidemic fever during the winter months, of which the reports furnish many examples, is an argument against the abstract effect of heat. Moisture, abstractedly considered as a cause of disease, is met by similar arguments. British Guiana has more rain by one half than Jamaica, but the mortality among troops in the latter situation is twice as great as in the former. Were excess of moisture the cause of the excess of disease in the western hemisphere, the same effect should be expected from it in the east; but the Malabar coast, which is deluged with rain six months in the year, is generally the most healthy quarter in the Madras presidency. We would remark on this reasoning of the author, in the force of which we fully concur, that it is in accordance with the received medical opinions on the subject, according to which an absolutely wet surface is less noxious than one in the intermediate condition between wetness and dryness. This cannot be more forcibly illustrated than by the remark of travellers in Africa. When the rain begins to fall there, the emanations are so noxious that the inhabitants retire to their houses, and endeavour to exclude even the least access of air. As the rain continues, and the ground becomes thoroughly wetted, the sickness abates, to be renewed with greater violence on the retiring of the rains and the ground becoming dry.*

The reporter, however, finally concludes that there is an influence in the production of sickness in the conjoined operation of heat and moisture. After stating that numerous instances have occurred in which, of adjacent islands, or even contiguous stations of the same island, subject of course in an equal degree to the operation of these agencies, the one has been desolated with fever, whilst the other has enjoyed a degree of salubrity equal to that of Great Britain, he yet finds on an average of a series of years (though not uniformly or equally in each year), that the greatest number of admissions into hospital and deaths take place in those months when the greatest degree of heat is combined with the greatest moisture.

The varieties in the physical and geological character of the soil the author does not regard as throwing any light on the subject under investigation. At many stations where the soil is exactly the same, the rate of mortality is very different; and on the other hand, where soils differ, the mortalities accord. While the soil and the physical characters, he remarks, are the same in every year, the sickness and mortality are extremely variable, and only in certain seasons attain an extraordinary degree of intensity. This remark is true, not only of the West Indies, but of every other climate in the world. We should have expected so philosophic a writer to have sought an explanation only of the ordinary state of insalubrity of any given district in its permanent condition. Epidemic prevalence is something extrinsic and superadded; and however glad we should have been to derive from a sta-

* Cyclopædia of Practical Medicine, article Malaria and Miasma, vol. iii. p 61.

tistical, or any source, an explanation of these visitations, we cannot help thinking this argument derived from them misplaced, when it is sought to be applied to the connexion, real or supposed, between the physical and geological character of the soil and the rate of sickness and mortality.

To the agency of marshes in generating fever, Major Tulloch attaches less importance than the majority of his professional readers, we are convinced, will expect. He says:

"That the vicinity of marshes, swamps, and lagoons, is generally subject to fevers, both of the intermittent and remittent type, is a fact sufficiently established by multiplied experience, both in tropical countries and within the temperate zones. But that remittents or yellow fever may be generated where no such cause is in operation to produce it; and that, consequently, it is impossible to establish a necessary connexion between this cause and the appearance of that disease, is sufficiently established by the fact, that the sickness and mortality in British Guiana and Honduras, where swamps and marshes most abound, are considerably less than at Up-Park Camp, and several other stations in Jamaica, remote from the operations of any such agencies." (*West Indian Report*, p. 102.)

This extreme insalubrity of certain of the Jamaica stations, especially that of Up-Park Camp, it is extremely difficult, or rather impossible, to explain upon principles ordinarily received regarding the generation of the class of fevers which produce the mortality there. This latter station is described as being about two miles north of the sea-port of Kingston, and lying at the eastern extremity of a well-cultivated and fertile plain, having a graduated slope towards the sea. About a mile to the north is a mountain ridge, the foot of which is slightly wooded, and four or five miles to the eastward extends another of greater elevation, but of which the surface is clear and open. There is no marshy or swampy ground nearer than three or four miles, and the soil is of so absorbent a nature, that it is necessary to dig to a considerable depth before water can be procured. The camp is elevated 200 feet above the level of the sea, and enjoys the advantage of a regular sea and land breeze, the former during the day and the latter during the night, which reduces the temperature considerably below what prevails at most of the other stations. The buildings occupied by the troops are raised on arches, well ventilated, and consist of three ranges, those for the officers in front, for the men in the centre, and for the hospital in the rear. The barracks are of brick, two stories high, the hospital of the same material, but of one story and a basement, with balconies all round.

Notwithstanding these apparent advantages, the mortality has been considerably above the average of this unhealthy island, being in the ratio of 140·6 per thousand, of the mean strength annually on an average of 20 years. This mortality is principally attributable to fevers, 120·8 per thousand of the mean strength having perished annually from this cause alone.

All the circumstances stated respecting Maroon Town, one of the healthiest stations, not only in Jamaica, but in the West India colonies generally, would, according to received opinions, threaten a much greater mortality than Up-Park Camp, if we except the greater elevation and more moderate temperature of the former station, for this post is situated on an elevation of upwards of 2,000 feet above the level of the sea, and the thermometer seldom rises higher than 80°, and is sometimes at night in winter at 52°. Being surrounded by high mountains, the climate is variable, and the temperature liable to sudden transitions. There is much rain, and the evaporation subsequently caused by a tropical sun produces frequent and dense fogs. The soil is a deep red clayey loam, extremely tenacious, rendering it almost impracticable to walk out for some hours after a shower, and retaining moisture for a very considerable time. Yet at this station the actual mortality has not exceeded 22 per thousand of the force annually, being the same as among the foot-guards in London on the average of the last seven years.

The circumstance to which Maroon Town owes its salubrity appears to be the great elevation and low temperature; for though there may be periods when the wetness of the climate and the impermeable nature of the soil may place the surface in a state of moisture beyond what is favorable to the production of malaria, yet there must be comparatively dry periods when such a locality, but for its elevation, would be pestilential in the extreme. This subject of elevation is shown to be a very important one, and fortunately it is one on which Major Tulloch and Sir A. Halliday are generally in accordance. The researches of Humboldt have shown that yellow fever is never known beyond the height of 2,500 feet; and Major Tulloch shows that at an elevation of from 2,000 to 2,500 feet, troops are likely to be wholly exempt from fever, or to encounter it in so very modified a form, that the mortality from all causes will not, on an average of a series of years, materially exceed that to which an equal number of European troops would be subject in the capital of their native country. He adds, very happily, that the diseases of the tropics seem, like the vegetable productions of the same regions, to be restricted to certain altitudes and particular degrees of temperature. Sir A. Halliday agrees generally with these views, but qualifies them by stating that an airy, *isolated* mountain in the neighbourhood of a pestilential marsh will always be more unhealthy than a marsh itself. He questions if a single mountain, rising out of a marshy plain, would be quite free from the influence of the poison, at even the elevation of 2,500 feet, but is certain that, as regards a range of mountains, that the third from the marsh will prove quite healthy at a much lower elevation than that fixed upon by Humboldt, and even if it is considerably lower than the two that intervene. We attach considerable importance to this statement, proceeding as it does from a man of Sir A. Halliday's observation and experience, and we would remark generally that this writer's observations on the localities to be selected as stations for troops in the West Indies indicate deep study and profound knowledge of the subject.

Sir A. Halliday adverts to certain sources of sickness and mortality which are left untouched in the Report, being indeed such as only a professional man could discern: one is deficient experience in the medical men in these colonies, arising from the constant changes consequent on the want of all inducement, either in the shape of colonial allowances or the prospect of promotion, to remain in so noisome a climate; vacancies, when they occur, being filled up from home, not from those in the station, medical men may be said rather to pass through these colonies than remain in them, and hence the soldiers are attended in sickness by a succession of inexperienced officers. The evils of this system, if it is correctly described by the author, must be very great; for in the epidemics or endemics of any given country the practitioner is useless, or worse, if inexperienced. The tone in which another supposed source of sickness and mortality is dwelt upon by Sir A. Halliday is creditable to his feelings as a man and a Christian. Believing that one half of the sickness and mortality which prevail among the troops arises from drunkenness and other immoralities, he forcibly and consistently contends for an extension of the present very deficient means of religious care and instruction, for the purpose of mitigating these evils. There is only one military chaplain for the whole Windward and Leeward Island command, and he is stationed in an island, Trinidad. There is in the different garrisons a church-parade every Sunday morning, "where some clergyman of the colony reads over, in a hurried manner, the prayers of the morning service, or perhaps only a part of them, and of what he does utter, few, if any, of the soldiers can hear one word." This mere congregational ministration, performed in the manner described by Sir A. Halliday, will probably be destitute of all influence, or certainly possess an influence very inferior to that of the more intimate and searching communications for which the seclusion of the hospital and the season of sickness and distress furnish the most fitting opportunities. Such communications between clergymen and soldiers cannot take place under the existing circumstances of the different garrisons; and it is suggested that in various stations there should be two regularly-commissioned chaplains, a Protestant and a Roman Catholic. In favour of the latter appointment, he quotes a very impressive case, the force of which and of the author's advocacy of the measure we strongly feel; but we can tell him, that were government to act on his just and well-intentioned suggestion, a fearful outcry would be raised by a numerous and powerful party in the state, that they were employing their influence, and spending the wealth of the country for the propagation of error on a subject regarding which, above all others, only truth should be taught. We mention this not in censure of the proposal abstractedly—for why should not the poor Catholic soldier have the consolation of that form of Christianity which alone can reach his heart?—but to point out to him the difference of position between irresponsible advisers and responsible actors.

As a means of diminishing the mortality in the West Indies, Sir A. Halliday calls the attention of the secretary-at-war and the public to Dr. Carl Warburg's fever-drops. We do not object to anything Sir Andrew says on the subject. He has expressed, as he was bound to do, his conviction of the efficacy of this medicine in a disease over which the reports before us prove but too distinctly that our power has hitherto been very limited. We cannot, however, avoid a degree of prejudice against secret nostrums; and the weight of miscellaneous testimony adduced in advertisements which meet our eyes in various quarters, serves only, by calling up unpleasant reminiscences and associations, to render this feeling more intense in the case of the present medicine. But our conviction being that the knowledge of the real pathology of fever is still very imperfect, and that the modifications of our remedial means derived from this imperfect pathology have tended little, if at all, to increase our power over the fevers, either of the West Indies or any other climate, we do not feel justified in rejecting assistance, in however questionable a shape it may present itself. The matter is a public one, and to government we think belongs the office, in the first place, of obtaining testimony, which should be unquestionable in character and ample in amount, of the efficacy of the medicine, and if such is obtained, of purchasing the secret for the public good. The station of Great Britain among nations, and particularly the extent of her colonial possessions render her the Power to which the investigation and the purchase properly belong; and for the former purpose her military hospitals in various quarters of the world furnish ready means, whilst, we are convinced, the different medical charities throughout this country (for Dr. Warburg informs us that it is a certain remedy for fever of any type) would cooperate in a work of general beneficence.

There is nothing more interesting in these Reports than the statistics of pulmonary diseases. They appear to us of great importance in reference to the etiology of these formidable maladies, by showing at least the error of certain views still prevailing on the subject, and by confirming, if they have not the merit of originating, more correct opinions. After a table showing the number of admissions and deaths from this class of diseases, in the Windward and Leeward Command, Major Tulloch remarks: "Though the proportion of admissions by this class of diseases is lower than among troops in the United Kingdom, in the proportion of 115 to 148, the ratio of mortality is much higher, as nearly 10½ per thousand of the strength have been cut off annually; whereas, in Britain, the deaths from the same class of diseases do not average at the utmost, more than 8½ per thousand. This arises from the *greater prevalence of consumption*, for out of an aggregate strength of 86,661 serving in the Windward and Leeward Command, not fewer than 1,023 were attacked by that fatal disease, being 12 per thousand annually, whilst out of an aggregate strength of 44,611 dragoon-guards and dragoons serving in Great Britain, only 286 were attacked, being about 5½ per thousand." "Not only," he adds, "is consumption productive of great mortality in this command, but inflammation of the lungs and chronic catarrh are nearly twice as prevalent and twice as fatal as among troops in Britain, thus showing how little effect a mere increase of temperature has in modifying these diseases." (*West Indian Report*, p. 8.) Major Tulloch might have extended his remark to uniformity of temperature; for this great mortality from consumption and inflammation of the pulmonary organs has taken place in a command where the greatest annual range in the thermometer is 13°, and in some parts of which it is only 4°.

In Barbadoes the mortality from diseases of the lungs is considerably above the very high average of the command, being 15·8 per thousand annually of the white and 18·7 per thousand of the black troops. It may not be amiss to remark that, whilst this report shows the general rate of mortality from fever to be considerably lower among the negroes than the whites, the former are found to suffer in a much greater proportion from consumption and other diseases of the lungs. In Jamaica pulmonary diseases are by one third less prevalent, and one third less fatal than in the Windward and Leeward Command. The mortality from them is estimated in the Report at 7·5 per thousand annually. The author remarks that, exclusive of those invalided on account of these diseases in the island and who died in their passage home or shortly after their arrival, this class of diseases has produced almost the same annual ratio of mortality as among the dragoon-guards and dragoons in the United Kingdom on the average of the last seven years. Consumption is, however, much more prevalent in Jamaica than Britain; for, whilst in that island 13 per thousand of the whole force have been annually treated for this disease, at home those who have undergone treatment on account of it have amounted only to between 5 and 6 per thousand annually, although the period over which the latter observations extend includes two severe epidemics of influenza, which probably laid the foundation of more cases of this disease than usually occur in this country. The author adds:

"That this fact is the more remarkable, as in Jamaica catarrhal affections are not one half so common as in Britain. Out of an aggregate strength of 51,567, there occurred but 2,809 cases, including both acute and chronic, or 65 per thousand of the strength annually; whereas, in this country, out of an aggregate strength of 44,611, no less than 5,462 cases are recorded, or 122 per thousand annually. Inflammation of the lungs is still more rare. The baneful influence of the climate of the West Indies in accelerating the progress of consumption has long been remarked by medical authorities; but it does not seem to have occurred to them, nor indeed had they any means of ascertaining, that at least twice as many cases originate in that climate as at home, though those catarrhal affections to which they are generally attributed are there comparatively so rare." (*West India Report*, p. 47.)

We find, then, in Jamaica, with a high temperature, that catarrh and inflammation of the lungs are rare, whilst consumption is twice as frequent as in Britain. The confirmation this affords of the opinion of Louis, and its refutation of that of Broussais are too evident to require to be indicated. These statistical details have an important bearing, too, on an opinion promulgated by the late Dr. Wells, that there was a natural antagonism between diseases, the product of marshy effluvia and consumption—that the one excluded the other. In the West Indies, however, we see the prevalence of the two diseases, supposed to exclude each other, coinciding.

The portion of the reports from the Mediterranean bearing on the same subject, contains much interesting matter. At Gibraltar we find that the deaths from diseases of the lungs in general amount to 5·3 per thousand of the average force, whilst the mortality from consumption alone amounts to 3·5 per thousand. Major Tulloch presents us with the following commentary on the return:

"The ratio of admissions by this class of diseases is to that in the United Kingdom as 141 in 148, the principal difference being that catarrhal affections are less frequent in Gibraltar, while inflammation of the lungs is much more so; the cases of the latter are, however, of a milder character, as only 1 in 45 died of those admitted into hospital in Gibraltar, while 1 in 18 died of those admitted for the same cause among the dragoon-guards and dragoons in the United Kingdom. The total mortality by diseases of the lungs would appear to be less at this station than at home; but that, we apprehend, arises from many of the consumptive patients being invalided, who, if they die on their passage or after their arrival in England, are not included in the returns of the station where their diseases originated. That this is sufficient to account for the difference may be supposed from the fact stated in the Medical Report for

1835, that during the thirteen years previous the average number of deaths from consumption in Gibraltar was $12\frac{3}{10}$ annually, besides about five sent home labouring under the same disease, of whom few or none recovered." (*United Kingdom and Mediterranean Report*, p. 11, *a*.)

The mortality from diseases of the lungs in Malta has been in the ratio of six per thousand during a period of twenty years. The author gives the following important commentary on the table containing the details:

"The climate of this island appears from the preceding results to be by no means favorable to persons predisposed to these diseases; the mortality is higher than in Gibraltar, and there is every reason to believe that, could we have taken into account the number invalided, and who died on the passage, it would have proved even higher than at home. It is somewhat remarkable that, in a climate where the thermometer never sinks to the freezing point, where the temperature at night is generally within a few degrees the same as during the day, and where those sudden transitions from heat to cold, to which this class of diseases is generally attributed in other countries, are extremely rare, the ratio of admissions should be only about one fifth less than in the United Kingdom. It may serve as a striking illustration how little influence the climate of Malta is likely to have in diminishing the tendency to consumption, that the proportion attacked by that disease among the troops there during the last seven years has averaged $6\frac{7}{10}$ per thousand of the strength annually, while in the United Kingdom, during the same period, the proportion attacked of the dragoon-guards and dragoons was but $6\frac{4}{10}$ per thousand annually." (*United Kingdom and Mediterranean Report*, p. 24.)

The author adds some remarks to show that the fatal influence of diseases of the lungs is not confined to the troops alone, but extends in a corresponding degree to the inhabitants. He refers to returns which prove that 6,664 deaths have occurred in 13 years from this class of diseases, constituting a mortality of 513 annually, which, upon an average population of 100,000 of all ages, is about $5\frac{1}{8}$ per thousand of the strength, being scarcely one per thousand less than among the troops, notwithstanding the night exposure of the latter in the course of their military duties. He adds that, though the climate of this island has been supposed favorable to diseases of the lungs, its inhabitants appear to suffer from them nearly as much as those of high northern latitudes, for the returns of Sweden show that there were only 14,087 deaths from this class of diseases out of the whole population in one year, being in the ratio of $5\frac{6}{10}$ per thousand, or within a fraction the same as in Malta.

In the Ionian Islands, the death, from all diseases of the lungs are 4·8 per thousand of the mean strength annually. Major Tulloch makes the following remarks on these numbers:

"Notwithstanding the variable character of the climate, the rapid alternations of temperature, and the tempestuous weather which frequently prevails in this command, diseases of the lungs are both less prevalent and less fatal than at Malta or Gibraltar: the admissions into hospitals being respectively as 90, 120, and 141, and the deaths as 4·8, 6·0, and 5·3 per thousand of the strength annually. The principal exemption in the Ionian Islands is from catarrhal affections, which are not half so prevalent or half so productive of mortality as in the other Mediterranean stations, or in the United Kingdom. Most of the deaths arise from consumption, but neither is the proportion attacked so high nor are the fatal cases so numerous as in Malta, where there exists a comparatively equable temperature, and that mild condition of the atmosphere which is supposed favorable to persons predisposed to that disease.

In Malta, on the average of twenty years, about 6 per thousand of the troops have been attacked annually by consumption; and in Gibraltar and the United Kingdom, nearly the same ratio; while in the Ionian Islands only 5 per thousand have been attacked, and the deaths have been fewer in the same proportion." (*United Kingdom and Mediterranean Report*, p. 35, *a.*)

When we pursue the subject through the reports from British America, we observe the same discrepancy between ordinarily-received opinions and statistical facts as has been displayed in the results of observation in other quarters of the globe. In Bermudas, with great uniformity of climate, and an absence of those extremes of cold to which such diseases in northern latitudes are frequently ascribed, we find inflammation of the lungs and consumption decidedly prevalent, and the mortality from pulmonary disease 8·7 per thousand annually, which is higher than among troops in the United Kingdom and the Mediterranean stations. In Nova Scotia and New Brunswick, with severe winters and sudden atmospherical vicissitudes, we find diseases of the lungs less prevalent than in the United Kingdom, in the proportion of 125 to 148, and less fatal in the proportion of 7·1 to 7·7. In Canada, distinguished for the severity of its winters, and so remarkable for sudden alternations of temperature that the thermometer has been known to fall at Quebec 70° in twelve hours; the admissions for pulmonary disease are 148, and the deaths 6·7 per thousand, the latter being much lower than in the United Kingdom. Major Tulloch points out the following striking facts, which require no comment: At Bermuda, there have been attacked annually by consumption, of every thousand, 8·8; in Gibraltar, 6·5; and in Canada, 6·5.

Major Tulloch modestly observes, that his object in the Reports is rather to state effects than to speculate on causes. We admit that he has been more successful in the case of pulmonary diseases, in showing what are not their causes than what are; but we feel, too, that in the state of our knowledge respecting these diseases, the former knowledge is a necessary and very important preliminary to the latter. One set of facts, however, in these Reports has an important bearing on another. We find between the classes, officers and soldiers, the most perfect equality exists as to mortality from fever, whence the reasonable inference is, that the cause of fever is general—that it is in the climate. But in the case of bowel complaints and diseases of the lungs, there is the greatest discrepancy in the extent to which these classes are respectively affected. From the former disease, the soldiers suffered in comparison of the officers in the proportion of nine to one, where, for five days in the week, the diet of the soldiers consisted of salt provisions; in colonies, on the other hand, where such provisions were issued to the troops only two days in the week, the mortality in the two ranks from these diseases approximates so closely as to be nearly on a par. Here is evidence almost demonstrative, which is further confirmed by the fact stated by Sir A. Halliday, that the fresh-meat rations, supplied to the soldiers at the request of Lord Howick, have effected a great diminution in the prevalence of bowel complaints. The relative prevalence and fatality of consumption in the two classes is very disproportionate; for in the Windward and Leeward command, the proportion of officers and men treated for the disease stands as 6 to 15; and in the Jamaica command, as 4 to 15; whilst the deaths among officers are, in the former station, one fourth, and in the latter one fifth, of what occurs among the troops generally.

To what is this prodigious discrepancy to be attributed? The author declines hazarding a positive opinion; but he refers to the views of Sir James Clark (and we are happy to observe his respectable and respectful reference to the labours of this truly enlightened physician), that improper diet and impure air are the most certain exciting causes of consumption among those not hereditarily predisposed to it, and to the experiments cited by him, which have proved that tubercular affections may be induced in animals by confinement in close humid places and innutritious food. Major Tulloch deems it consequently not improbable that crowded barrack-rooms and a restriction to salt-diet may, particularly in a tropical climate, produce a similar effect on the constitution of soldiers.

Sir A. Halliday, though he admits the *predisposing* influence of the causes assigned by Major Tulloch, thinks that the exciting cause of the prevalence of pulmonary disease among the soldiers is to be found in the barracks being generally seated on some lofty eminence. The officers' journeys, he says, to and from these stations, are less frequent than those of the soldiers, and are performed in a less hurried manner; the latter, from overstaying their leave, being obliged to hasten to barracks, and this too when they are heated by indulgence in the grog-shops. We do not feel satisfied with this *rider* to Major Tulloch's very reasonable deduction, thinking the arguments adduced in its support, anything rather than conclusive. For instance, we find the following statement: "The troops at Antigua are principally quartered on a range of heights about 400 feet above the level of the sea, commanding the entrance to English harbour. The dryness of the atmosphere in this island is also remarkable; and hence we find that the ratio of deaths from diseases of the lungs is as high as nine per thousand." Now, besides that the argument here is so illogically stated, that we are at a loss to know whether the "range of heights," or "the dryness of the atmosphere," is the source to which the *high* rate of mortality is traced; we find, that in the Windward and Leeward Island command, in which Antigua is situated, the average mortality from pulmonary disease is ten per thousand, so that the heights or the dryness, as the case may be, reduce instead of augmenting the mortality.

For only one other subject contained in these Reports (desirous though we are to put the reader into as full possession as possible of their contents) can we at present find space in our pages. This subject is the sickness and mortality among troops in the United Kingdom. We shall not detail the reasons which have induced the reporter to confine his examination to the casualties of the household troops and dragoons; suffice it to say, that these forces alone can furnish correct information as to the influence of the climate of Great Britain on the health of soldiers.

Among dragoon guards and dragoons, the admissions into hospitals amount to 929 per thousand annually, and the deaths in hospitals to 14 per thousand; and if we add to this latter number the deaths from suicide, murder, drowning, and other casualties, amounting to $1\frac{3}{10}$ per thousand, we have an annual mortality of $15\frac{3}{10}$ per thousand of the mean strength. The estimate was formed from a period, during a portion of which cholera and influenza prevailed, and 2 per thousand will be a reasonable deduction for the excess of mortality produced by these diseases: consequently $13\frac{3}{10}$ per thousand will form the average mortality. The age of the soldiers constituting this force averages from 29 to 30. A fair estimate of the mortality among persons of the same age in civil life shows it to be at the rate of 11·5 per thousand annually.

This is a great discrepancy, and of a kind to indicate that the military profession, even under the most favorable circumstances, operates prejudicially to the health of those employed in it. It should be remembered, however, that the estimate of those in civil life is drawn from the kingdom generally, which furnishes a much lower rate than would be formed were the estimate deduced from the civil population of towns, in which, be it remarked, the troops are quartered. The mortality of an urban population in civil life, of the same age as the soldiers, is 16 per thousand annually; it hence appears that the apparent high ratio among the troops, as compared with the general mass of the population, arises not so much from any deteriorating influence in their profession as from the disadvantage of their being subject to the insalubrious atmosphere of densely-populated districts.

In the foot-guards the ratio of mortality is 21·6 per thousand of the mean strength annually, or nearly one half higher than in the dragoon-guards and dragoons, and this excess of mortality arises entirely from diseases of the lungs. These troops are principally, as is known, stationed in the metropolis; but the conclusion that this striking excess is produced by the climate of London would be inadmissible, for it is fully shown that the climate of London is not more insalubrious than that of many other towns in which the troops are quartered throughout the kingdom. When the mortality among other classes exposed to the influence of a metropolitan atmosphere is examined, we find it in much lower ratio than that of the foot-guards. The average annual mortality of the civil population, between the ages of 20 and 40, is 15 per thousand; that of the East India Company's labourers 12½ per thousand, at the same period of life; and that of the household cavalry, likewise exposed to the climate of the metropolis, is 14·5, or not so high by one half as among the foot-guards.

It is stated that diseases of the lungs are the source of the prodigious excess of mortality in these troops, as compared with other classes of the military in this country. The following numbers show this statement to be correct. Of the dragoon-guards and dragoons there die annually, from diseases of the lungs, 7·7 per thousand; of the household cavalry, 8·1; of the soldiers of the West Indian depôts, which are stationed in England, a proportion of whom have been in the West Indies, and are likely to have received some contamination of their constitution from a climate tending to induce consumption, 9·6; of the foot-guards, 14·1. When the civil population is selected for comparison, the same preponderance is manifest; for, by calculations deduced from the London bills of mortality from 1830 to 1835, it has been ascertained that out of a thousand deaths among the civil population, the number by diseases of the lungs were 328, or scarcely one third of the whole; whereas, out of

745 deaths among the foot-guards, no less than 487, or upwards of two thirds, were from these diseases.

Major Tulloch offers no explanation of the extreme prevalence of this class of diseases in the guards, beyond the remark that it "originates in some peculiarity in the moral or physical condition of that description of troops, from which the others are comparatively exempt." In this view all must concur. The excess cannot depend on the climate of England or even in London; it lies within the very narrow sphere of the guards themselves, and its cause might, we think, be ascertained without much difficulty; and it is material that it should; for, in a minor degree, this excess of diseases of the lungs pervades the army at home generally when it is compared with the civil population; and it seems probable that the whole is dependent on some common cause, which exists in its greatest intensity in the guards. The report informs us that "nearly four fifths of the fatal cases of diseases of the lungs arise from consumption, being as many as from all other causes in the army at home. The highest estimates in civil life rate the mortality from this disease at a seventh part of the deaths of all ages; or, if the observation is confined to adults alone, it may possibly amount to a fourth part, being at the utmost only half as high as among the troops."

The congregation of soldiers into barracks is the main distinction, which can be supposed to affect their health prejudicially, between them and individuals of the same station in society around them; and to crowded barracks, and consequent atmospheric impurity, we are strongly disposed to ascribe the proneness to consumption among our troops. We cannot now enlarge on this opinion, or on certain reasons, extrinsic to the disease now under consideration, we have for holding it. That Major Tulloch's suspicions, at least, take the same direction is manifest from the following remark:

"From the very extensive calculations of Mons. Lombard, on the influence of professions on consumption at Geneva, it has been found that persons whose occupations were of an active description, inducing muscular exercise, and carried on in the open air, were not half so liable to that disease as those whose occupations were sedentary or carried on in shops and manufactories. If the aggregation of a number of men into one apartment, even though the space is not very confined, creates a tendency to this disease, then it clearly points out the propriety of affording the soldier as ample barrack accommodation as possible, not with a view to his comfort alone, for that is a matter of minor consideration, but in order to check the ravages of a disease which creates more mortality than all the others to which troops in this country are subject." (*United Kingdom Report*, p. 13.)

We now take leave of these Reports, with the opinion we formed of their value from a first perusal enhanced by further attention to their contents. They constitute a rich repository of facts from which practical lessons may be immediately drawn, and to which many an enquirer will turn in a future day, and invariably with profit. The pamphlet of Sir A. Halliday is not so closely reasoned as the reports; it contains, also, some important errors, which have been pointed out by Major Tulloch since its publication; but it is, on the whole, a very creditable performance, and does honour to the zeal and good feeling of the author.

